# Permafrost and Freshwater Systems in the Arctic as Tipping Elements of the Climate System

**DOI:** 10.1007/s10712-025-09885-9

**Published:** 2025-05-02

**Authors:** Victor Brovkin, Annett Bartsch, Gustaf Hugelius, Elisa Calamita, J. Jelle Lever, Eunhan Goo, Hyungjun Kim, Tobias Stacke, Philipp de Vrese

**Affiliations:** 1 Max Planck Institute for Meteorology, 20146 Hamburg, Germany; 2 CEN, Universität Hamburg, 20146 Hamburg, Germany; 3 b. Geos GmbH, Industriestr., 1A, 2100 Korneuburg, Austria; 4 Department of Physical Geography and Bolin Centre for Climate Research, Stockholm University, 106 91 Stockholm, Sweden; 5 Swiss Federal Institute of Aquatic Science and Technology (Eawag), Überlandstrasse 133, 8600 Dübendorf, Switzerland; 6 Swiss Federal Institute for Forest, Snow and Landscape Research (WSL), Zürcherstrasse 111, 8903 Birmensdorf, Switzerland; 7 Korea Advanced Institute of Science and Technology, 291 Daehak-ro, Yuseong-gu, Daejeon, 34141 South Korea

**Keywords:** Permafrost, Lake, Hydrology, Earth observation, Modelling, Feedback, Tipping point

## Abstract

The Arctic is warming several times faster than the rest of the globe. Such Arctic amplification rapidly changes hydrometeorological conditions with consequences for the structuring of cold-adapted terrestrial and aquatic ecosystems. Arctic ecosystems, which have a relatively small buffering capacity, are particularly susceptible to hydrometeorological regime shifts thus frequently undergo system-scale transitions. Abrupt ecosystem changes are often triggered by disturbances and extreme events that shift the ecosystem state beyond its buffering threshold capacity thus irreversibly changing its functioning (ecosystem tipping). The tipping depends on spatial and temporal scales. At the local scale, feedback between soil organic matter and soil physics could lead to multiple steady states and a tipping from high to low soil carbon storages. On the continental scale, local tipping is smoothed and the changes are rather gradual (no clear tipping threshold). However, due to the centennial timescale of soil carbon and vegetation dynamics, Arctic ecosystems are not in equilibrium with the changing climate, so a tipping could occur at a later time. Earth Observation (EO) is useful for monitoring ongoing changes in permafrost and freshwater systems, in particular extreme events and disturbances, as indicators of a possible tipping point. Lake change observations support gradual rather than abrupt transitions in different permafrost regions until a hydrological tipping point where lake areas start to decline leading to regional drying. Due to floodplain abundance, floodplains should be considered separately when using satellite-derived water extent records to analyse potential tipping behaviour associated with lakes. Reduction in surface water extent, increasing autocorrelation of water level of larger lakes and the impact of extreme events on ground ice can all be observed with satellite data across the Arctic. The analysis of Earth System simulations suggests significant impacts of changes in permafrost hydrology on hydroclimate in the tropics and subtropics, but there is no clear threshold in global temperature for these shifts in hydroclimate.

**Article Highlights**

- The presence of multiple steady states in permafrost systems suggests the possibility of local tipping of ecosystems and soil carbon storages, albeit at centennial rather than decadal scales
- Earth Observation (EO) of extreme events and disturbances is useful for monitoring ongoing changes in permafrost and freshwater systems as indicators of a possible tipping point
- The area of EO-derived open water in the Arctic both increases and decreases with a tendency of the net effect being towards a smaller area. For conclusions regarding thaw lake dynamics and tipping, floodplains should be considered separately
- An analysis of timeseries of lake water levels suggests that lake hydrology is shifting state within permafrost regions
- Earth System simulations suggest changes in permafrost hydrology can have significant effects on the hydroclimate in the tropics and subtropics, but the changes are gradual rather than abrupt

## Introduction

Permafrost is an important component of the cryosphere. It is formally defined as ground (soil or rock) frozen for at least two consecutive years. Permafrost underlies about 14 million km^2^ in the Northern Hemisphere (Obu 2021) with its largest areas in Russia, Canada, the USA, and China (Sun et al. 2018; Wu et al. 2021; Zhao et al. 2017). The deepest permafrost exists in Central Siberia, where it is more than a kilometre thick (Yershov 1990) because that region was not glaciated during the last glacial cycle (Lindgren et al. 2016) and therefore exposed to extremely cold temperatures at the surface. Cold and frozen conditions in permafrost-affected soils slow down the decomposition of soil organic matter. Organic matter accumulates in the upper layer and remains undecomposed for millennia (van Huissteden 2020). Permafrost-affected soils and sediments of the Northern Hemisphere contain 1,100 to 1500 Pg of organic carbon (Hugelius et al. 2014), about twice as much as the pre-industrial atmosphere in 1850. This soil organic matter is strongly affected by anthropogenic warming and could be released to the atmosphere as CO_2_ or CH_4_, well-mixed greenhouse gases (GHGs), amplifying climate change (permafrost carbon feedback, Schuur et al. 2015). To date the Arctic has warmed almost four times faster than the rest of the globe (Rantanen et al. 2022). While at present the net permafrost carbon fluxes are not very pronounced and the net permafrost region CO_2_ budget is near neutral (Bruhwiler et al. 2021; Hugelius et al. 2024), this could change in the future as the current rate of warming and thaw is unprecedented in the last few thousand years (Schuur et al. 2022)*.* Re-formation of thawed permafrost carbon is such a slow process that it makes these changes practically irreversible on human (multidecadal) timescales (de Vrese et al. 2023a). This is why a number of recent studies proposed the permafrost system as a tipping element in the Earth System (Armstrong McKay et al. 2022; Wang et al. 2023). In this paper, we focus on 1) quantitative characteristics of permafrost and freshwater systems (such as carbon or ice content, lake area) which are susceptible to hydrometeorological regime shifts thus which frequently undergo system-scale transitions; and 2) observations of these characteristics using remote sensing or modelling using process-based understanding. We follow the recent IPCC definitions of tipping element, irreversibility, and abruptness of climate changes (Box 1). These definitions are rather broad. For example, a tipping point could be interpreted as a point in time after which the system reacts much faster than before to an external forcing (abrupt change), or as a shift in the ecosystem state towards a different mode of functioning, which may be slow but irreversible (ecosystem tipping). In this paper, a tipping element is characterised by a large spatial scale that is visible on a planetary level (Armstrong McKay et al. 2022; Wang et al. 2023). Our preferred definition of tipping includes nonlinearity in response to environmental changes and/or irreversibility of changes on human (multidecadal) timescales.

- The presence of multiple steady states in permafrost systems suggests the possibility of local tipping of ecosystems and soil carbon storages, albeit at centennial rather than decadal scales
- Earth Observation (EO) of extreme events and disturbances is useful for monitoring ongoing changes in permafrost and freshwater systems as indicators of a possible tipping point
- The area of EO-derived open water in the Arctic both increases and decreases with a tendency of the net effect being towards a smaller area. For conclusions regarding thaw lake dynamics and tipping, floodplains should be considered separately
- An analysis of timeseries of lake water levels suggests that lake hydrology is shifting state within permafrost regions
- Earth System simulations suggest changes in permafrost hydrology can have significant effects on the hydroclimate in the tropics and subtropics, but the changes are gradual rather than abrupt

**Box 1:**
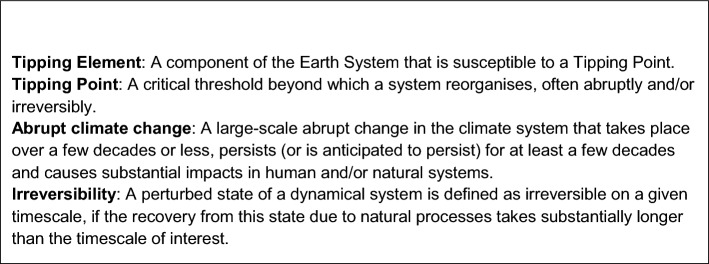
Definitions of tipping terms after AR6 WGI Glossary (IPCC, 2021).

One argument that supports permafrost as a tipping element comes from high-resolution ice-core records. During the last deglaciation, the atmospheric CO_2_ concentration increased abruptly 3 times with amplitudes of 10–12 ppm in each case (Marcott et al. 2014). These changes on the centennial scale occurred simultaneously to large warming events in the Earth system, presumably due to reinvigoration of the Atlantic Meridional Overturning or AMOC (Brovkin et al. 2021). The estimated amount of carbon released during each of these events is about 50 PgC (Kohler et al. 2014). The timescale of abrupt carbon increase points to the land source rather than the ocean which has a larger storage capacity but a slower response. At the peak of the Last Glacial Maximum (LGM, around 21 thousand years ago) the northern permafrost region was about 13 million km^2^ larger than today, including 5 million km^2^ on what are today shallow polar sea shelves (Lindgren et al. 2016). Lindgren et al. (2018) estimated that the total soil carbon stock in the LGM permafrost region was ca. 2400 PgC. Importantly, the LGM carbon stocks were dominated by permanently frozen carbon, while the present soil carbon is predominantly stored in seasonally frozen soils. The reconstructions support postglacial reductions in the permafrost carbon stock of around 1000 PgC (Lindgren et al. 2018). The abrupt release due to the simultaneous thawing of carbon frozen in the permafrost in response to abrupt warming is a plausible explanation of the geological record, indicating that it cannot be excluded in the future.

In the Arctic, organic carbon processing is controlled not only by temperature but also by hydrology with increase in GHG emissions from the inland freshwaters (lakes and rivers) (Ramage et al. 2024). The fractional area of lakes in the high latitudes of the Northern hemisphere is higher than elsewhere on the globe (Lehner and Döll 2004) though surface water extent has been decreasing across the Arctic (Webb et al. 2022). Many of these lakes are so-called thermokarst lakes (Grosse 2013), formed when the melting of massive ground ice bodies causes surface subsidence and formation of lakes in the depressions. These water bodies may increase the transport of thermal energy into the permafrost, leading to formation of year-round unfrozen ground layers (taliks) below these lakes (Burn 2002) as well as lateral erosion of lake shores (Arp et al. 2011). Significant areas of the high northern latitudes are covered by former thermokarst lake basins (Bergstedt et al. 2021; Grosse 2013; Jones et al. 2022).

The outgassing of CO_2_ and CH_4_ from lakes represents a significant component of the global carbon cycle (Lauerwald 2023b). Lakes in permafrost regions contribute to these emissions (Serikova et al. 2019; Wik et al. 2016). The organic carbon released from thawing permafrost is partly labile and thus it can be mineralised and emitted from these lakes to the atmosphere in the form of CO_2_ and CH_4_. A small fraction of the organic carbon from thawing permafrost is released into rivers and, consequently, into the ocean. About 15–20% of dissolved and particulate organic matter (DOC and POC) in Arctic freshwater systems originates from permafrost or peat (Vonk et al. 2015; Wild et al. 2019). Seasonal changes in the direction of flow may be of importance, and while the Arctic Ocean constitutes 1% of the world’s ocean volume, recent estimates indicate that the Arctic Ocean receives 13% of the terrigenous DOC load delivered to the world’s oceans (Liu et al. 2024; Raymond et al. 2007; Stein and Macdonald 2004), which is proportional to the pan-Arctic watershed fraction of 14% of the global land area (McGuire et al. 2009).

In addition to the land permafrost area, about 2–2.75 million km^2^ of permafrost is present on the Arctic shelf seafloor; this permafrost was submerged over the last 18 thousand years during and following the deglaciation. This subaquatic permafrost stores another 2800 (1518–4982) PgC (Miesner et al. 2023) in addition to that stored in terrestrial permafrost. Subaquatic permafrost temperatures are higher than their terrestrial counterparts while the sea water temperatures are around 0 °C almost the whole year. This makes thawing at the top of subsea permafrost extremely slow, with timescales being from centuries to millennia, while the main thawing occurs at the bottom of the permafrost column due to geothermal heat. Since the next glacial period is expected to be at least 50,000 years from now (Archer and Ganopolski 2005; Berger and Loutre 2002), the subsea permafrost is expected to thaw completely within the next few millennia (Wilkenskjeld et al. 2022). Most of organic matter of terrestrial and marine origin in the subsea permafrost will be decomposed and transported as CO_2_ or CH_4_ through the sediment layer to the ocean water column on a millennial timescale (Puglini et al. 2020) and then to the atmosphere, amplifying anthropogenic warming.

The paper is structured as follows. Section 2 describes the changes in the Arctic permafrost and freshwater systems associated with tipping. In Sect. 2.1, we discuss abrupt changes, nonlinearity and the irreversibility of permafrost thawing processes and hypothesise that abrupt hydrological changes could lead to a local tipping point (nonlinearity) in the transient response, but will not propagate to a nonlinear instantaneous response at the pan-Arctic scale. In Sect. 2.2, we discuss observations of extreme weather events that could lead to abrupt permafrost changes. In Sect. 2.3, we continue by analysing trends in lake characteristics in permafrost regions, noting that high losses of water area are often associated with floodplains. In Sect. 2.4, we analyse early warning signals in the permafrost lake level time series and hypothesise about signals of regime shifts in lake hydrology. In the Sect. 3, we discuss a possible future shift in global hydroclimate in response to hydrological changes in the permafrost region, that could affect other Earth tipping elements such as the Amazon rainforest and the vegetation of the Sahel. We conclude in the Sect. 4.

## Changes in Permafrost and Freshwater Systems in the Arctic

### Abrupt Changes, Nonlinearity, and Irreversibility of Permafrost Thaw Processes

Permafrost thawing processes occur at several different scales, either as a landscape-wide successive increase in seasonal surface thaw (active layer deepening) or through localised abrupt surface collapse (thermokarst) caused by the melting of excess ground ice (ice that exceeds the volume of soil pore space). Thermokarst often leads to the formation of distinct landform types, including different types of thaw lakes, post-thaw peatlands and thaw slumps on hill slopes. Because permafrost that is rich in ground ice is often simultaneously rich in organic carbon, thermokarst-prone terrain holds about half of the permafrost carbon stock despite covering only 20% of the permafrost region surface area (Olefeldt et al. 2016). Thermokarst formation via abrupt thaw processes, triggered by loss of ground ice, are not yet included in global or regional scale models, but can lead to significant increases in post-thaw greenhouse gas fluxes (Turetsky et al. 2020). Under high emission scenarios, abrupt thaw carbon losses are projected to account for approximately 40% of the mean net emissions attributed to widespread gradual thaw by 2300, even if they affect only a small surface area (Turetsky et al. 2020). At policy relevant timescales, abrupt thaw processes can also be considered irreversible, as the loss of ground ice and the surface deformation it causes is (semi)permanent even if the permafrost should refreeze. It can thus be considered as a local-scale tipping element within the permafrost system.

**Fig. 1:**
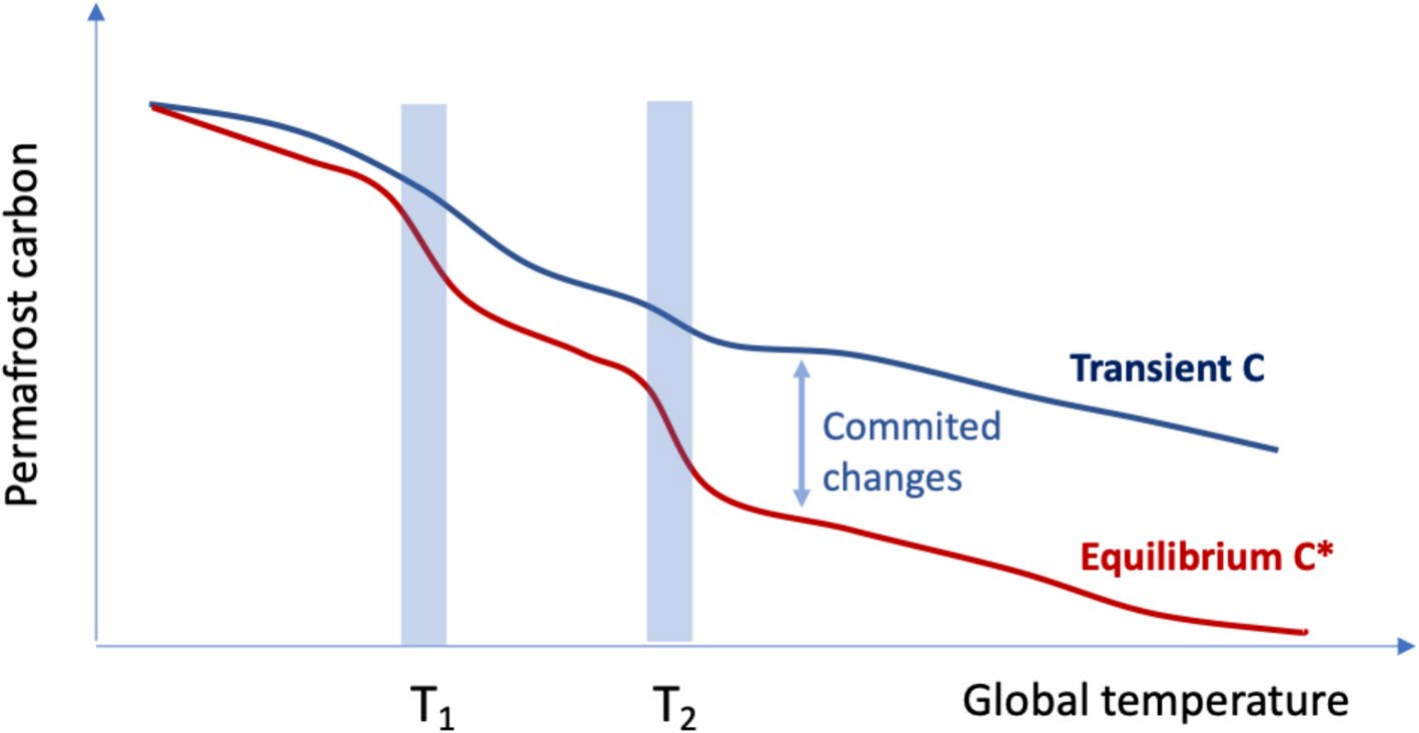
Conceptual plot of permafrost carbon storage as a function of global temperature. C* is the equilibrium carbon storage for a given global temperature, C is the time-dependent storage (at the time of reaching T), C–C* is the committed carbon thaw. Due to spatially heterogeneous climate changes and permafrost carbon storages, there are hypothetical nonlinearities at the tipping points T_1_ and T_2_ which could be monitored by Earth Observation (EO)

To identify tipping points in permafrost carbon dynamics, we search for nonlinearities in the permafrost system response to changes in environmental conditions. Because the permafrost thaw and carbon decomposition combine fast and slow processes, we need to account for a range of different timescales. The carbon dynamics are different for transient responses, such as simulated by Earth System models in future scenarios with a defined end point, e.g. the year 2100 (Nitzbon et al. 2024), and for committed changes occurring in a quasi-equilibrium response arrived at after a substantial delay (Fig. 1). Areas with larger carbon storage will have larger carbon decomposition on multidecadal to centennial timescales. Therefore, our hypothesis is that the equilibrium response is more nonlinear than the transient one. Note that the existence of nonlinearities doesn’t imply the existence of multiple states in the permafrost carbon system.

Irreversibility of changes in permafrost and freshwater systems depends on timescale. Processes in permafrost-affected soils exhibit a high level of inertia in their response to changing climate conditions and many may not be reversible or even stoppable on centennial timescales (Wang et al. 2023). Rising carbon emissions from recently thawed ground are a prominent example of such committed changes (Comyn-Platt et al. 2018; Gasser et al. 2018; Natali et al. 2021). These do not only notably reduce the remaining carbon budget of the more ambitious climate goals but may even hold the potential to lead to a self-sustaining warming after all man-made greenhouse gas emissions have ceased (Randers and Goluke 2021). One reason for this inertia are the extremely long carbon turnover times which imply that it could take centuries for soil carbon uptake and emissions to reach a new steady state (Shu et al. 2020; Wu et al. 2021). The same is true for the physical state of the subsurface, where the large amounts of energy either required for or released in the phase change of water could mean that trends in soil temperatures and the soil water and ice content continue long after the climate has stabilised (de Vrese and Brovkin 2021; Eliseev et al. 2014). This latter point is also highly relevant for the surface hydrology as many of the lakes and wetlands in permafrost-affected regions depend on microtopographic features caused by ice wedges. The continued thawing of ground ice could be leading to subsidence and changes in topography (Aas et al. 2019; Ekici et al. 2019; Nitzbon et al. 2021) and thus the current trends in lake and wetland cover (Webb and Liljedahl 2023). These trends could continue even after the climate stabilises due to the hysteresis of the hydrological response to climate change (de Vrese and Brovkin 2021).

A number of these change processes could be halted if global heating and the temperature trend was partially reversed in a temperature overshoot scenario (Schwinger et al. 2022). However, the warming during the overshoot temporarily allows the decomposition of formerly frozen organic matter, thus, reducing the soil carbon content. Given that soil carbon stores and excess ice have built up over thousands of years, a reversal of the impacts of a temporary warming may still take centuries if not millennia. In addition, simulations with land surface models including permafrost indicate that parts of the Arctic and subarctic zone may exhibit different stable steady states depending on the soil organic matter concentration at the time of climate stabilisation (de Vrese and Brovkin 2021). Therefore, even if global temperatures return to pre-industrial levels, some land areas may never return to the soil carbon stocks that existed before anthropogenic warming.

The separation between instantaneous and committed response is typical for systems with both short and long response timescales such as permafrost. In particular, the hydrological response of the land surface on local scale could be fast. With warmer and longer summers, the hydrology of the permafrost regions is changing. Ground ice is melting, new thermokarst lakes are forming, while older lakes drain and disappear (Grosse, 2013). Our working hypothesis is that abrupt hydrological changes could lead to a local tipping point (nonlinearity) in the transient response, but it will not propagate to a nonlinear instantaneous response on the pan-Arctic scale.

### Extreme Weather Events as Drivers of Abrupt Permafrost Changes

With increasing warming, extreme events and disturbances such as heatwaves, floods, fires and rain-on-snow are occurring more often (Romanou et al. 2024). All extremes potentially contribute to permafrost thaw and the mobilisation of carbon. The ground warms following rain-on-snow events (Westermann et al. 2011) while ground ice content is altered, for example, after flooding (Zwieback et al. 2023) or fires (Michaelides et al. 2019). However, extremes effect on land carbon components including vegetation biomass, soil organic carbon, and sediments in freshwater systems is not well quantified. EO through use of satellite data can potentially support the identification and quantifications of the impact of extremes and eventually allow the estimation of the scale of carbon and climate consequences of extremes.

**Fig. 2:**
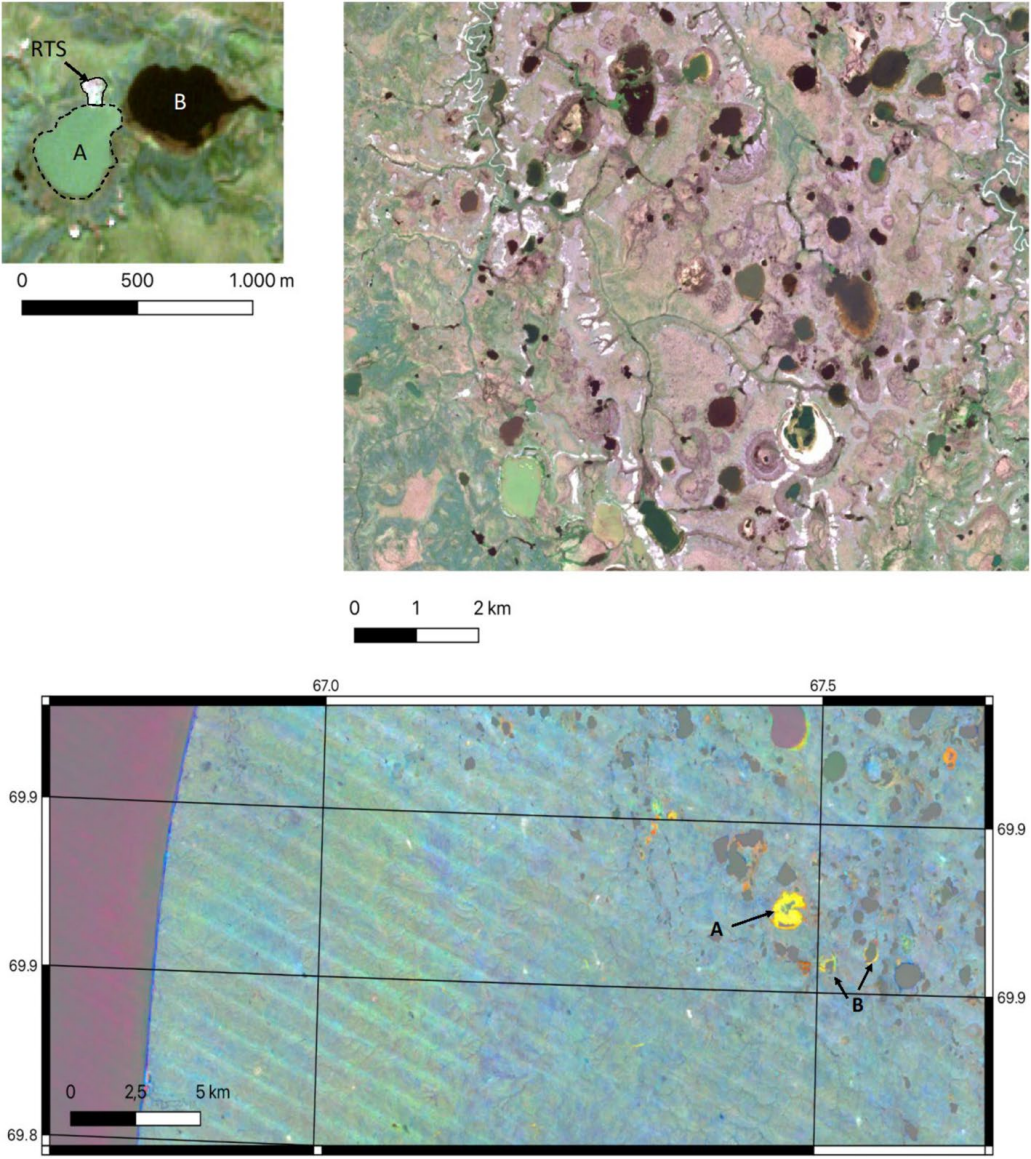
Remote sensing images of abrupt thaw. **Top**: Subsets of a red–green–blue composite of an Copernicus Sentinel-2 image from central Yamal on 23.07.2017: *top left* – Example of active thaw slump (RTS, bright feature, boundary indicated through black solid line), with impact on lake colour (soil material in suspension in left lake, boundary indicated through black dashed line), compared to neighbouring lake. *Right* – Clusters of thaw lakes in different states of drainage. **Bottom**: Landsat trend visualisation for central Yamal (1999–2014, source:Nitze 2018) – the blue band north to south on the left side indicates coastal erosion, the yellow patch and circular features on the right side indicate lake drainage (A – almost complete, B – partial shrinkage with exposure at lake margins)

For example, heatwaves have been identified as a trigger for retrogressive thaw slumps (RTS) across the Arctic including in Canada (Jones et al. 2019; Kokelj et al. 2017; Lewkowicz and Way 2019), Alaska (Balser et al. 2014) and in Russia (Babkina et al. 2019; Bernhard et al. 2022; Kizyakov, 2013; Runge et al. 2022). RTS are a thermokarst feature that are a consequence of melting ground ice (also called cryogenic landslide (Burn and Lewkowicz 1990)) and are classified as abrupt permafrost thaw features (Turetsky et al. 2020). Melting ice lenses or ice wedges at the base of the active layer leads to ground collapse and eventually slope failure. In-situ observations for selected thaw slumps are available and sufficient data to describe the relation to unusually warm summers can be obtained through satellite data (Lewkowicz and Way 2019) primarily using Landsat data with 30 m spatial resolution (Fig. 2). RTS formation leads to vegetation removal which allows identification using the multispectral observations. For example, more than 4000 thaw slumps have been initiated since 1984 in an area of 70.000 km^2^ (Lewkowicz and Way 2019), covering an area of 64 km^2^ according to analyses of Landsat data. Detection capability has been recently significantly improved with the availability of Sentinel-2 with 10 m nominal resolution (Lissak et al. 2020). However, even better resolution of fine detail including topographic changes would be required for precise quantification of the amount of soil which is mobilised by RTS.

Permafrost degradation leading to loss of ground ice after extreme events such as heatwaves, flood and fires could be measured from space. Subtle changes in the order of centimetres can be captured through Synthetic Aperture Radar (SAR) data. Increased subsidence in anomalously warm summers was for example described by Bartsch et al. (2019) for central Yamal in Russia. The magnitude of the increase differed depending on soil types. Specifically the melting of tabular ground ice at the base of the active layer has been suggested to be related to warm extremes. Zwieback et al. (2021) studied this for a case on the Alaskan North Slope using actual ground ice in-situ data. Subsidence can be also triggered by flooding events (Zwieback et al. 2023). Wildfires remove vegetation and part of the surface organic layer, deepen the active layer, thaw the ground ice and increase seasonal subsidence (Michaelides et al. 2019). An increased subsidence was observed using the SAR data for more than a decade after burn activity (Michaelides et al. 2019). Overall, ground subsidence monitoring can capture the consequences of weather extremes but spatially and temporally consistent records, which are basic requirements for tipping analyses, are not available to date (Bartsch et al. 2023a). Subsidence monitoring may nevertheless support regional studies of hydrological changes and associated carbon flux alterations, but so far, most investigations in permafrost environments have focussed on technical retrieval challenges (Widhalm et al. 2025).

Gas emission craters, which are associated with warm years, have been shown to transform into lakes. Large craters are known from the Yamal Peninsula (e.g. Kizyakov, 2015). Monitoring of their formation process requires highly detailed terrain information. Pingo-like mounds form beforehand, which have been documented with repeat stereo photogrammetry (Kizyakov 2015). Lake formation and further growth as a consequence of such craters can be quantified with satellite data. Terrain and landcover changes can be however also associated with other processes and the attribution of their cause is challenging.

### Changes of Lakes Properties in Permafrost Regions

In general, several physical, chemical or biological processes that may pave the way for abrupt transitions to alternative states can be identified for lakes (Calamita et al. 2024). Some of these shifts may, in turn, have strong feedbacks to the climate system. An example of such a regime shift is the abrupt change in lake water level which affects lake extent and potentially leads to lake shrinking (Zhao et al. 2017) or lake formation (Shugar et al. 2020; Stuart-Smith et al. 2021; Sun et al. 2018). Permafrost thaw, accelerating due to climate change, can create or expand thermokarst lakes with impacts on hydrology, ecology and biogeochemistry (in 't Zandt et al. 2020). In particular, carbon stored in lake sediments could be decomposed leading to the release of both CO_2_ and CH_4_ (in 't Zandt et al. 2020). Thus, climate change, by increasing the length of the ice-free season increases the annual water body GHG emissions at high latitudes (Wik et al. 2016). However, the quantification of GHG emissions from lakes is hampered by high uncertainties due to limited spatial and temporal coverage of observations and uncertainties in the quantification of inland water surface areas (Lauerwald et al. 2023a), especially in the case of thermokarst lakes (Olefeldt et al. 2016). While the fluxes can be estimated by data-driven estimates of the gas budget of the permafrost region (Ramage et al. 2024), the feedback of such inland waters on the climate system is not yet represented in Earth System Models (Turetsky et al. 2020).

EO has only been used to a limited extent for detecting and studying tipping points and regime shifts in lakes, especially compared to in-situ measurements, paleolimnological records or models (Calamita et al. 2024). So far, EO data has been primarily applied to detecting abrupt changes in lake water extent (Calamita et al. 2024). However, EO offers significant advantages for monitoring inland waters, serving as a strategic tool for assessing and tracking lake quality worldwide through frequent, large-scale surveys (Giardino et al. 2014). While most key variables for monitoring tipping systems are classified as Essential Climate Variables (ECVs) of the Global Climate Observing System (GCOS), some critical lake properties—such as particulate organic carbon and chlorophyll-a—remain unaccounted for (Loriani et al. 2025).

A range of remotely sensed datasets has been published recently that allow the assessment of land surface hydrology changes in permafrost regions, specifically lakes. However, no dataset is currently available which can address changes in permafrost thaw lakes comprehensively. Current climate data records are limited to large lakes or are not specifically for lakes. Both these issues can be attributed to the lack of spatial resolution (Bartsch et al. 2023b). Parameter retrievals include lake surface temperatures (e.g. LakeCCI (v2.0.1, Carrea, 2022; Carrea et al. 2023)), lake water level (GLWS v1.1, Yao et al. 2023) and change in fractional water area (e.g. by Webb et al. (2022)). Such records allow the determination of trends and in some cases application of techniques for identification of tipping, such as the analyses of temporal autocorrelation (TAC) in time series. Current land surface temperature missions which provide time series at a global scale include for example MODIS (500 m) and Sentinel-3 SLSTR (1 km). Both use thermal sensors which leads to data gaps in case of cloud cover. An alternative are passive microwave observations, which provide data only at the tens of kilometre scale. Future thermal missions such as LSTM (Land Surface Temperature Mission, Sentinel program) will provide records with better than 100 m detail, but the frequent Arctic cloud cover remains an issue (Bartsch et al. 2025). Water level can be obtained through altimeter, however, these also provide only coarse footprint sizes of several hundred metres. Any climate data record analysis for LST and water level is therefore only feasible for larger lakes. However, such larger lakes are typically of a different origin to permafrost thaw lakes. Observed variations can, however, serve as a general indicator of increasing air temperatures and changes in the hydrological cycle.

Water surface extent can be derived independently from monitoring of specific lake objects therefore long-term analyses based on coarse spatial resolution observations is feasible (e.g. Schroeder et al. 2015). However, identification of thaw lake change and attribution to abruptness requires higher spatial and temporal resolution. Recent and future satellite missions may provide relevant data but sensors used in the past had only limited capabilities. Available circumpolar long-term change studies of surface water extent do not separate lakes from floodplains. Extensive floodplains are spread across the Arctic which regionally dominate land surface hydrology change patterns and surface water extent varies considerably seasonally (Bartsch et al. 2012; Watts et al. 2014). A multitude of lakes exists which also shows strong seasonality although not closely located to river courses (e.g. Bartsch et al. 2012; Trofaier et al. 2013). The fluvial patterns overlap with thermokarst related changes of surface water extent.

**Fig. 3:**
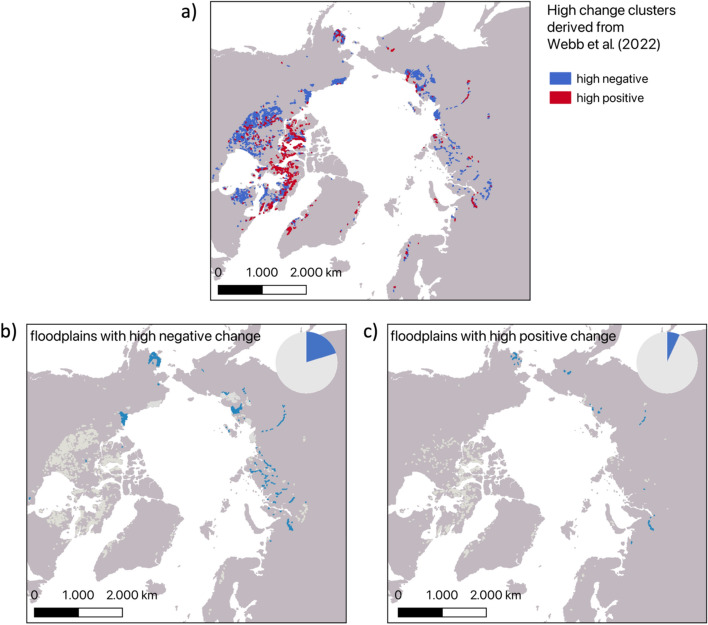
Superfine water index (SWI) change (2000–2021) attribution to river floodplains. a) Clusters with SWI decline (high negative) versus increase (high positive) extracted from Webb (2022). b) Distribution (blue areas) and proportion of cluster areas with high negative trends within river floodplains. c) Distribution (blue areas) and proportion of cluster areas with high positive trends within river floodplains

The use of trends based on the superfine water index (SWI) derived from multispectral information at 500 m (MODIS) as suggested nevertheless provides a step forward compared to the coarser global water fraction datasets based on microwave records. The SWI is positively correlated with percentage surface water cover. In the study by Webb et al. (2022), widespread water area loss across the Arctic for the last two decades (2000–2021) was found. Webb et al. (2022) further differentiated between various regions considering ground ice content, permafrost zonation and lake cover percentage. The water surface shrank on average in all of these categories. However, here too, lakes were not separated from floodplains. Due to the high spatial detail available from MODIS, an estimate of the split between these might be feasible. Smaller lakes cannot be separated within pixels but river floodplain areas can be attributed to specific pixels due to their larger extent. In order to demonstrate this we created a subset of the SWI trend dataset (Webb 2022) based on several geospatial datasets which represent non-floodplain and floodplain areas (Fig. 3). We first derived regional clusters with strong positive and negative SWI trends (areas with high change) by the following procedure: 1) extraction of pixels where SWI trends exceed one standard deviation (> ± 0.005), 2) polygonalisation and application of a 2 km buffer and 3) extraction of clusters larger than 5 km^2^. These clusters were semi-automatically assigned to floodplains or non-floodplains by considering terrain information (Copernicus Digital Elevation Model slope), the circumpolar land cover dataset of (Bartsch et al. 2024) and Google hybrid visualisations. Major coastal plains (northern part of the Alaskan North Slope, Lena Delta, Yana, Nenets reserve and Kanin coastal plain) were defined as none-floodplain. The proportion of floodplain regions which underwent high losses for 2000–2021 was 20% (and 15.5% of all areas with high change) (Fig. 3b). 7% of the areas with high water fraction gain (and 1.5% of all areas with high change) were part of floodplains. In total, at least 17% of change is floodplain-related. This needs to be considered when satellite-derived water extent records are used to analyse potential tipping behaviour linked to lakes.

Significant seasonal variations have been documented for selected Arctic deltas (Vulis et al. 2020) as well as smaller lake sizes in case of warmer climate conditions (Vulis et al. 2021). More precise accounts including actual seasonality change identification across the entire Arctic, separately for lakes in typical thermokarst landscapes and water bodies in floodplains, are needed to create datasets suitable for tipping behaviour analyses. This requires the use of as high spatial and temporal resolution as possible based on SAR data, which is unaffected by clouds. Seasonal patterns can be identified on a regional scale if sufficient data is available for selected years (example for Russia: Reschke et al. 2012). However, recent SAR missions have so far not provided regular consistent coverage of the Arctic land area (Bartsch et al. 2023b). This can be attributed to differences in acquisition strategies across the globe, partially driven by sensor failure (as for example in case of Copernicus Sentinel-1B). The recent launch of Sentinel-1C is, however, expected to advance circumpolar-scale studies. Locally, high spatial resolution optical data can also provide insight into seasonality (Cooley et al. 2019; Mullen et al. 2023). Such observations may serve as quality control for circumpolar assessments.

### Emerging Signals of Regime Shifts in Lake Hydrology

Global lakes have responded dramatically to climate change in recent decades, including water level (Woolway and Merchant 2019). Lake water level (LWL) is recognised as an Essential Climate Variable (ECV) by the Global Climate Observing System (GCOS) and is a fundamental variable to understand the balance between water inputs and water loss in lakes. In the presence of disruptions in Lake Hydrology, substantial changes in Lake water level and surface water extent will occur. In particular, in ice-rich permafrost regions, increased surface temperature and ice cover loss can alter the water and energy balance in lakes. Such changes can lead to corresponding feedback mechanisms in regional to local hydrological or biogeochemical cycles.

When a complex system is becoming unstable, short-term responses to weak and continuous perturbations will slow down, thus slowing the recovery of the system back to the original state. Theoretical studies have demonstrated that these phenomena can be precursors to critical transitions as the system approaches a tipping point (Scheffer et al. 2009). This is also referred to as critical slowing down (CSD). During CSD, the increased memory in the time series, which can be quantified with increased temporal autocorrelation (TAC), has been proposed as an indicator of declining resilience (Held and Kleinen 2004; Scheffer et al. 2009). Here, resilience is defined as the capacity of the system to remain in its current state despite external perturbations.

There has been a widespread change in global lake water storage. Arctic lakes have mostly been in decline, attributed to a combination of changes in precipitation, runoff, temperature and potential evapotranspiration (PET) (Yao et al. 2023). This aligns with other observations that show the reduction of lake area across the northern permafrost zone through surface and subsurface drainage or lake infilling (Webb and Liljedahl 2023; Webb et al. 2022). On the other hand, in the Tibetan Plateau, glacier retreat and permafrost thawing has driven alpine lake expansion (Yao et al. 2023). This is consistent with the recent trends of rapidly increasing lake volumes in the Tibetan Plateau where the majority of the additional water supply is from increased net precipitation (74%), followed by glacier mass loss (13%), and ground ice melt due to permafrost degradation (12%) (Zhang et al. 2017). Along with these observations, we provide additional analysis of the signals of the hydrological system of the lakes in the permafrost zone consistently losing resilience. To explore the regime shifts in Lake Hydrology and how it has evolved over the last decade, we present here the TAC time series estimated from remotely sensed lake water levels.

Combining multiple satellite altimetry sensors, the Global database of Lake Water Storage (GLWS v1.1, Yao et al. 2023) provides information on surface areas, water levels, and storage for 1972 of Earth’s large water bodies spanning from 1992 to 2020 at a monthly frequency. From the globally distributed lakes, we selected 97 lakes located in the permafrost regions (permafrost probability > 0.5) of the Northern Hemisphere. This includes both the continuous and discontinuous permafrost regions where recent findings (e.g. Webb and Liljedahl 2023; Webb et al. 2022) have demonstrated surface water decline, especially in discontinuous permafrost regions (permafrost probability 0.5–0.9). Of these, 69 lakes were selected from the Northern Arctic, and 28 from the Tibetan Plateau. A lake showing spurious drop in water level, attributed to dam failure, was removed (Liu et al. 2016). To obtain Lake water level anomalies, we first removed the long-term linear trend by performing a simple linear regression on the monthly time series and subtracting the fitted trend from the original data. To capture interannual variability, we computed annual mean z-score anomalies based on monthly time series after removing seasonality, which ensures the analysis is robust, as the observations are only available during the boreal summer.

The 15-year (2006–2020) mean annual TAC time series of lake water level anomalies shows an increase, particularly since 2010 (Fig. 3a, c). To account for differing geographic regions and permafrost extents, we grouped the lakes into four categories based on combinations of region (i.e. Arctic or Tibetan Plateau) and permafrost probability (i.e. continuous or discontinuous) (Fig. 3b). In the Arctic lakes, both the continuous and discontinuous permafrost regions show an increasing TAC time series from 2010 (continuous: *τ* = 0.64, *P* = 0.006; discontinuous: *τ* = 0.53, *P* = 0.03). However, since there was also a decline in TAC values until 2010, we cannot clearly disentangle if the increasing and decreasing signals are due to internal natural variability or if they are fingerprints of climate change. Multidecadal oscillations affecting Arctic air temperature (Chylek et al. 2009; Fang et al. 2022) and alterations in thermal or mixing regimes in lakes may affect these temporal changes in TAC. In the Tibetan Plateau, trends of TAC time series for both the continuous and discontinuous permafrost regions are insignificant (*P* = 0.087). Unlike Arctic lakes, many lakes on the Tibetan Plateau seem to have already entered a transient phase, where lake water levels exhibit strongly increasing trends, and thus their TAC time series might not show significant trends in recent periods. Of the 95 lakes, 32 lakes (33.7%) exhibit positive trends, 10 lakes (10.5%) exhibit negative trends and 55 lakes (57.8%) show insignificant trends (Fig. 3d).

Our findings suggest that lake hydrology is shifting state within permafrost regions, which agrees with various regional studies (Andresen and Lougheed 2015; Liu et al., 2024a; Saros et al. 2025; Su et al. 2023). For example, sub-lake talik formation or expansion can accelerate permafrost thaw by thermokarst processes, and increased runoff can induce river-to-ocean heat transport, which will create a positive feedback loop in sea-ice loss (Nitzbon et al. 2024). Also, changes in snow cover on the Tibetan Plateau can dynamically influence climate variabilities in other regions (Li et al. 2018; Zhang et al. 2023a) and establish teleconnections to other tipping elements (Liu et al. 2023). As freshwater systems become more sensitive and approach tipping points, they can possibly trigger nonlinear responses, leading to widespread impacts on hydrology, ecology, and biogeochemistry (Anthony et al. 2018; de Vrese and Brovkin 2021; Hessen et al. 2024; Wunderling et al. 2024).

It should be noted that most of the lakes considered here are located outside the identified clusters with high surface water index change as derived from Webb et al. (2022) (Fig. 3). They are mostly glacially formed, larger lakes, outside of typical thermokarst regions. Altimeter data which can provide the lake levels for the relatively small lake sizes typical for thaw lakes, would be required to extend the above analyses to such small thaw lakes. The capabilities of state-of-the-art SAR-based altimeter data (e.g. Corpenicus Sentinel-3 with along-track footprint of 330 m) are still limited although an improvement.

## Remote Effects of the Permafrost Thaw on the Earth System

The feedback between permafrost and climate is usually considered by studying permafrost carbon emissions to the atmosphere in the form of well-mixed GHGs (Schuur et al., 2022). There is another way how permafrost changes could affect the climate, and this is through changes in land surface physics and hydrology (Bonan 2016). Increased permafrost thaw in the future is expected to lead to a shift in land surface hydrology towards greater subsurface flow and increased Arctic river discharge (Rawlins and Karmalkar 2024; Zhang et al. 2023b), affecting the oceanic freshwater budget and ocean circulation. The Arctic is a cloudy region (Chernokulsky and Esau 2019). The Arctic clouds have a strong impact on the surface energy budget, generally warming the surface in winter and cooling it in summer (Kay and L'Ecuyer 2013), altering the climate feedbacks (Goosse et al. 2018). During plant growth periods, evapotranspiration from plants leads to higher moisture fluxes to the atmosphere, more clouds, and less radiation reaching the surface. The presence of permafrost limits the amount of water that ca’ be transpired (van Huissteden 2020), and less transpiration means a less moist atmosphere and less clouds, warming the surface (Bonan 2016). Changes in Arctic vegetation affect climate through both transpiration and albedo changes (Swann et al. 2010). Such biogeophysical feedbacks from permafrost to climate have rarely been considered in analyses of the Earth System.

**Fig. 4:**
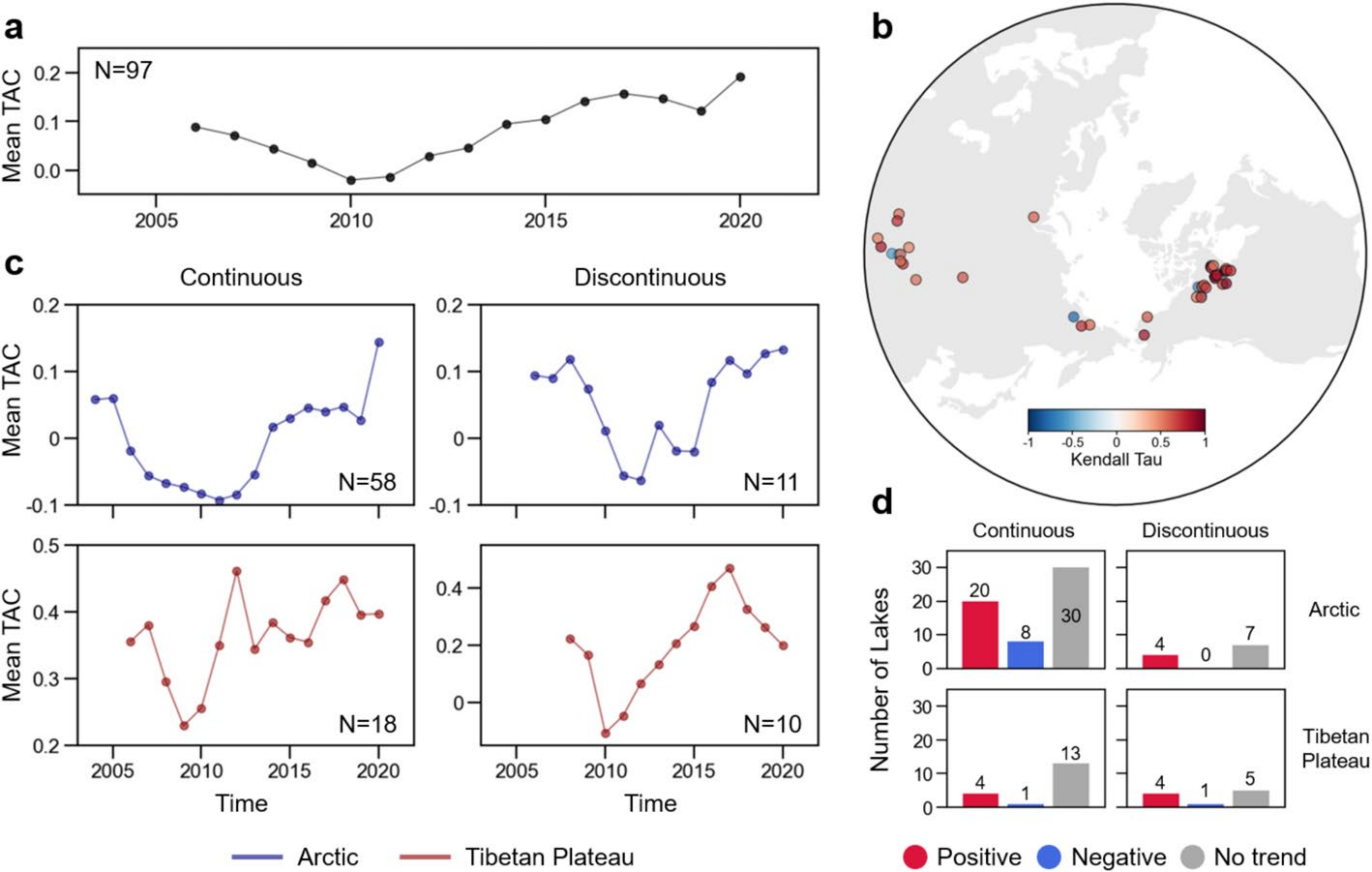
**a.** Mean TAC time series for lake water level anomalies calculated from every selected lake (N = 97). **b.** A circumpolar map of the Kendall tau (τ) values of each lake’s TAC time series from 2010 (*P* < 0.05). Insignificant lakes are not shown. **c.** Same as (a), but the lakes are divided into four categories: based on permafrost probability (continuous/discontinuous), and geographic regions (Arctic/Tibetan Plateau). Only the years having TAC values for more than 90% of the samples are shown for each case (**a.**, **c.**) **d.** Trends of TAC time series for selected lakes based on τ values from 2010 divided into four categories. Positive trends mean increasing TAC time series, which implies a decline in resilience, and vice versa for negative trends. No trend implies insignificant τ values

**Fig. 5:**
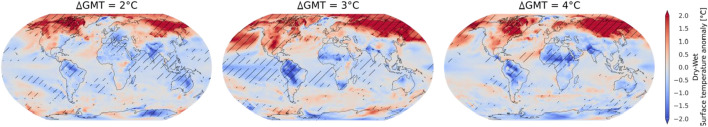
Remote effects of changes in permafrost hydrology on changes in global mean temperature, GMT (hatched areas indicate significant differences). Temperature differences between the Dry and Wet permafrost setups are shown for 2, 3, and 4 °C of global warming (± 0.25 °C). Data from (de Vrese et al. 2023b)

Recent analyses suggested that changes in energy and water cycling in the Arctic will have an effect far beyond the Arctic region, providing biogeophysical teleconnections to the other tipping elements such as Atlantic meridional overturning circulation, Amazon, or Sahel (de Vrese et al. 2023a) and through change in wetland distribution, to methane emissions from tropical regions (de Vrese et al. 2023b). In these studies, the hydrological modelling parameters in high latitudes were changed between the limits of a plausible range of parameters configurations, namely between the Dry and Wet setups (Fig. 4). In the Dry setup, enhanced drainage and runoff lead to a higher water flux to the ocean than to the atmosphere, resulting in less moisture recycling between land and atmosphere. In boreal summer, when the high latitudes receive almost the same cumulative radiations as the tropics, this leads to less cloud cover and a relatively warmer and drier land surface. In the Wet setup, more moisture was recycled between land and atmosphere, leading to a higher cloud fraction, and wetter and cooler conditions at the land surface in summer (de Vrese et al. 2023a). For Wet and Dry simulations using a coupled land–atmosphere-ocean model, the MPI Earth System model (MPI-ESM), run until 2100 under the RCP8.5 scenario of greenhouse and other forcings, it is observed that in both simulations the Arctic region got warmer, but with different regional patterns. For a valid comparison, the differences between the climate change patterns have to be taken at the same global temperature level. In Fig. 5, the regional patterns of differences in annual mean surface temperature are presented for 2, 3, and 4 °C global warming levels. Because the differences are taken at the same temperature level for both, the Dry and Wet simulations, the average change in the temperature for each plot is zero.

For both setups, the high northern latitudes show a strong warming especially due to lower cloud cover in summer, compensated by cooling elsewhere. The relative cooling over land in South America and North Africa is very pronounced and statistically significant (Fig. 5). This relative cooling for these two regions is especially strong at the 3 °C of global warming level. While there is some change in patterns between 2 and 3 °C warming levels, generally the difference between the Wet and Dry simulations remains of the same magnitude. Although there are some changes in spatial patterns with increasing forcing, they are gradual rather than abrupt. We can interpret this as a gradual tipping with increasing global temperature. Although the changes are gradual, they indicate the future hydrological regime in the permafrost region will have a strong impact on other parts of the climate system, including the Sahel and Amazon, which have themselves already been identified as sensitive tipping elements (McKay et al. 2022). The analysis of permafrost’s role as a tipping element in the climate system is going to be continued in the framework of the Tipping Point Modelling Intercomparison Project (TIPMIP) (Winkelmann et al., in preparation). Similarly, the role of the cloud physics in tipping elements of the climate system is studied in the Cloud Feedback Model Intercomparison Project (CFMIP) (Webb et al. 2017) with the aim of improving understanding of cloud–climate feedback mechanisms, in particular in the permafrost region (de Vrese et al. 2024). Together, model intercomparison projects help to understand linkages between different components of the Earth System and potential nonlinearities in the climate system dynamics.

## Summary and Conclusions

Permafrost in the Arctic was formed during the geological past and served as a storage of organic carbon during relatively cold phases. It could have released carbon during abrupt thaw events during the last deglaciation, contributing to the reconstructed increase in atmospheric CO_2_ and CH_4_ concentrations. Current understanding is that substantial amounts of permafrost carbon could be released to the atmosphere in the future in response to anthropogenic warming, amplifying climate change. This carbon release, as simulated by existing Earth System and land surface models, is rather gradual and proportional to ongoing warming. This does not qualify permafrost carbon to be a tipping element. However, many local feedbacks are neglected in ESMs. Existence of alternative steady states supports the possibility of ecosystem tipping, although on centennial rather than decadal scale. Study of the equilibrium response of permafrost carbon to climate change on centennial to millennial timescale has not yet been done, and the difference between the instantaneous and equilibrium response could be pronounced.

Hydrological processes in lakes have internal feedbacks, which can lead to nonlinear responses to ongoing anthropogenic warming. The area of lakes in the Arctic both increases and decreases with a tendency of the net effect towards a smaller area which means effective drying of the land surface. At the same time, there is an increase in autocorrelation in the amount of water in lakes, with a caveat that only data for large lakes is available. As an early warning signal of changes in the water level, increased autocorrelation could indicate potential shift towards another state, but a possibility remains that this change is an artefact of the method. Higher resolution data and driver analysis are needed for further clarification. Earth observation can potentially also offer the means to capture the impact of extreme events on ground ice and to study changes in land surface hydrology’s seasonality. Observation capabilities currently limit studies to a local to regional scale, however, the recently launched satellites such as Copernicus Sentinel-1C will help to enable circumpolar-scale studies.

To conclude, the crucial question about changes in the Arctic is whether the Arctic will be drier or wetter in the future. Using Earth System model simulations, we demonstrate that this difference in wetness is important for hydrometeorological changes far beyond Arctic. We have not identified nonlinearities in the climate response. However, we cannot rule out that even linear changes in climate will be translated into nonlinear changes in CO_2_ and CH_4_ emissions because of nonlinearities in the ecosystem response. In addition to the potential of permafrost and freshwater ecosystem systems to regime changes on local and regional scale, a possibility also exists for a nonlinear response on a pan-Arctic scale.
